# Circular RNAs in hepatitis B virus-induced hepatocellular carcinoma: A comprehensive review and recent advances

**DOI:** 10.1016/j.gendis.2025.101605

**Published:** 2025-03-18

**Authors:** Wenjun Quan, Kizito Eneye Bello, Rafidah Hanim Shueb, Nazri Mustaffa

**Affiliations:** a Department of Medicine, School of Medical Sciences, Universiti Sains Malaysia, Health Campus, Kubang Kerian, Kelantan 16150, Malaysia; b Department of Microbiology, Faculty of Natural Science, Kogi State University (Prince Abubakar Audu University), Anyigba, Kogi State 1008, Nigeria; c Department of Medical Microbiology and Parasitology, School of Medical Sciences, Universiti Sains Malaysia, Health Campus, Kubang Kerian, Kelantan 16150, Malaysia; d Institute for Research in Molecular Medicine (INFORMM), Universiti Sains Malaysia, Kubang Kerian, Kelantan 16150, Malaysia; e Hospital Universiti Sains Malaysia, Kubang Kerian, Kelantan 16150, Malaysia

**Keywords:** Biomarker, circRNA, Hepatitis B virus, Hepatocellular carcinoma, Therapy

## Abstract

Circular RNAs (circRNAs) are a class of stable and versatile non-coding RNAs that are pivotal in the occurrence and development of some diseases, particularly tumors. Hepatitis B virus (HBV)-induced hepatocellular carcinoma (HCC) is a liver disease with substantial global impact. Despite efforts towards adequate management, the survival of patients with HBV-induced HCC has been consistently low. circRNAs regulate various physiological activities of HBV-induced HCC. This review aims to elucidate the biogenesis of circRNAs and the pathophysiology of HBV-induced HCC and comprehensively analyze the applications of circRNAs in oncology and therapeutics. In addition, this review summarizes past research achievements on circRNAs in HBV-induced HCC. Finally, the limitations of existing methodologies and circRNA research in HBV-induced HCC have been discussed to provide a blueprint for future investigations.

## Introduction

Circular RNAs (circRNAs) were originally discovered in the 1970s as a byproduct of gene cleavage with no implications.^1^ However, with technological advancements such as high-throughput sequencing, the biosynthesis and functional properties of circRNAs have been extensively analyzed for their potential medical applications.^2^ circRNAs are a novel subclass of non-coding RNAs with no 5′-terminal cap and 3′-terminal poly (A) tail that are abundantly expressed in various cells, tissues, and microvesicles.^3^ circRNAs possess covalently closed structures formed from the exons of pre-mRNAs through back-splicing, making them more insensitive to exonucleases than those of linear RNAs. Lengths vary from several hundred to thousands of nucleotides, with an average of 547 nucleotides.^4^^,^^5^ The major subtype is the exonic circRNAs, which account for approximately 85% of all circRNAs, including exonic, intronic, exon-intron, intergenic, and antisense circRNAs.^6^, ^7^, ^8^ circRNAs function as miRNA sponges, protein scaffolds, transcriptional regulators, modulators of alternative splicing, RNA stabilizers, nuclear transporters, and translation templates.^9^, ^10^, ^11^ Moreover, circRNAs are commonly characterized by cell, tissue, disease, or sequence specificity and are highly stable, which is crucial for research on cancer, cardiovascular diseases, neurological, immunological disorders, and diabetes.^12^^,^^13^ circRNAs have potential implications for diagnosis and cancer therapeutics. Although numerous circRNAs have been identified, the processes by which they affect disease pathogenesis and development remain largely unknown.

The incidence of liver cancer is anticipated to increase by 55% between 2020 and 2040, with a possible 1.4 million people diagnosed in 2040 by age-standardized incidence rate.^14^ Hepatocellular carcinoma (HCC), which accounts for more than 7% of all liver cancers, is a major health concern because of its high morbidity and mortality rates among all malignancies. Various factors increase the risk of HCC, including hepatitis B virus (HBV) infection, hepatitis C virus infection, heavy alcohol consumption, diabetes mellitus, aflatoxin B1, aristocholic acid, metabolic associated fatty liver disease, and hereditary causes.^15^^,^^16^ HBV is the primary cause of HCC. HBV belongs to the family Hepadnaviridae and contains covalently closed circular DNA (cccDNA), which serves as a transcriptional template to generate circulating DNA virions and viral antigens.^17^ Because currently available antiviral drugs such as nucleoside analogs are less useful in preventing cccDNA-related viral replication, DNA polymerases are unable to proofread bases, leading to HBV mutations during viral replication, and the elimination of HBV in HBV-infected patients remains challenging.^18^ Moreover, mutations in the basal core promoter (BCP) region of HBV and HBV genotypes (especially genotypes B and C) are associated with HCC.^19^ Although the association between HBV and HCC has been documented previously, the mechanisms and treatment of HBV-induced HCC remain unknown. Furthermore, the efficacy of drugs for treating HBV-HCC remains suboptimal, and the overall prognosis for advanced liver cancer remains unfavorable. Therefore, developing novel therapeutic drugs and identifying valuable biomarkers to facilitate HCC treatment is imperative. Owing to their unique properties, circRNAs may act as therapeutic endpoints and therapeutic molecules, in addition to being biomarkers for HBV-HCC. Additionally, circRNAs are important modulators of HBV-HCC progression and pathogenesis; however, the underlying mechanisms remain unexplored. This review aims to explore the contributions of circRNAs to cancer and HBV-associated HCC and provide potential insights for future research directions.

## HBV progression into HCC

**Figure 1:**
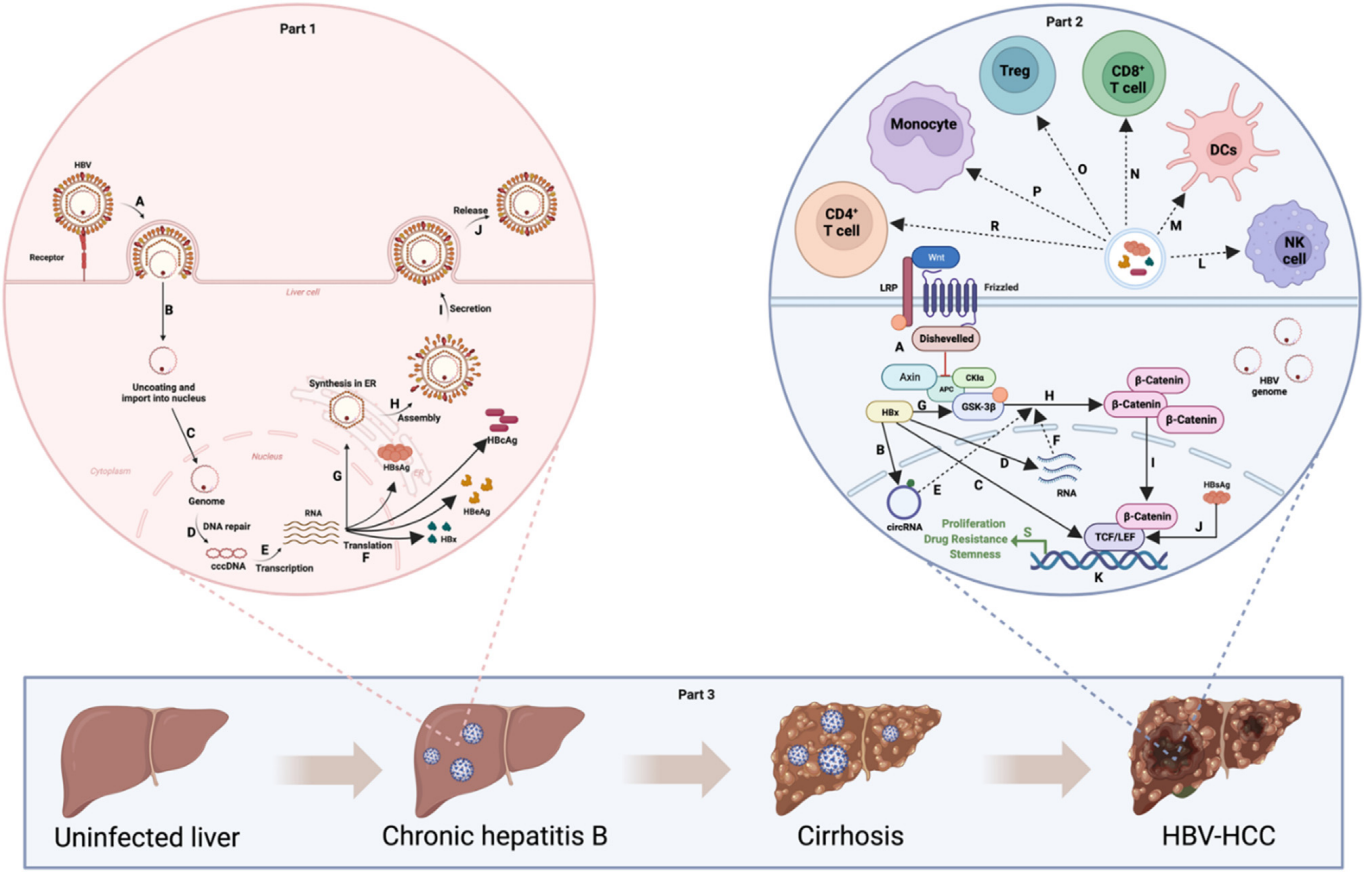
Part 1: The process of HBV infection and replication in cells: **(A)** HBV enters the liver cell via fusion and endocytosis. **(B, C)** The HBV genome is released into the cytoplasm and delivered to the nucleus. **(D**–**F)** Host proteins repair gaps in rcDNA to form cccDNA, which is transcribed into various RNAs, translating diverse proteins such as HBsAg, HBeAg, HBcAg, and HBx. **(G**–**J)** The multiple components assemble into an intact HBV released outside the cell. Part 2: The influence of proteins from HBV in HCC tumorigenesis: **(A)** Wnt combines with LRP and the frizzled receptor to inhibit the function of the Axin/APC/CK1α/GSK-3β complex by interacting with Dishevelled. The Axin/APC/CK1α/GSK-3β complex cannot phosphorylate β-catenin to promote its decomposition. **(B)** HBx mediates epigenetic modifications of RNAs, such as circRNAs. **(C)** HBx activates TCF in the Wnt/β-catenin signaling pathway. **(D)** HBx boosts RNA replication. **(E, F)** RNAs participate in the Wnt/β-catenin signaling pathway and activate the pathway. **(G**–**I)** HBx represses the function of the Axin/APC/CK1α/GSK-3β complex; therefore, β-catenin accumulates in the cytoplasm and enters the nucleus. **(J)** HBsAg increases the expression of LEF. **(K, S)** Multiple mechanisms facilitate the combination of β-catenin and TCF/LEF, regulating gene expression to promote the proliferation, drug resistance, and stemness of HBV-HCC cells. **(L**–**R)** The products, such as HBsAg, HBeAg, HBcAg, and HBx, from the HBV-infected HCC cells increase the expression of PD-1, CD244, CTLA4, IL-10, and PD-L1, and decrease the expression of IFN-γ, TNF-α, and activating receptors on many immune cells. Part 3: Four stages of HBV-HCC development: The process by which a healthy liver becomes HBV-HCC includes the following four stages: uninfected liver, chronic hepatitis B infection, cirrhosis, and HBV-HCC; however, a healthy liver does not progress to cirrhosis in all patients. HCC, hepatocellular carcinoma; HBV, hepatitis B virus; rcDNA, relaxed circular DNA; cccDNA, covalently closed circular DNA; HBeAg, hepatitis B virus e antigen; HBsAg, hepatitis B surface antigen; HBcAg, hepatitis B core antigen; HBx, HBV x protein; PD-1, programmed death-1; CTLA4, cytotoxic T-lymphocyte associated protein 4; IL-10, interleukin 10; PD-L1, programmed cell death ligand 1; IFN-γ, interferon-gamma; TNF-α, tumor necrosis factor alpha; APC, adenomatous polyposis coli; CK1α, casein kinase 1α; Axin, Axis inhibition protein; GSK-3β, glycogen synthase kinase-3β; TCF, T-cell factor; LEF, lymphoid enhancer factor.

Multiple factors, such as HBV products, HBV DNA integration, various non-coding RNAs, HBV mutations, epigenetic modifications, signaling pathways, host metabolism, intestinal microbiota, and immune components, contribute to the progression from HBV to HCC.^20^^,^^21^ Interestingly, the HBV x protein (HBx) is a vital HBV product that interacts with other factors to stimulate hepatocellular malignant transformation. HBx is an expression product of the HBx gene, which can activate oncogenes and repress tumor suppressor genes via genome integration. The integrated HBx gene has the potential to sustain the production of mutated and truncated HBx. HBx can modulate circRNAs, miRNAs, and other molecules through epigenetic modifications, such as N6-methyladenosine (m6A), to further influence signaling pathways, metabolic processes, and immune pathways related to the onset and development of HBV-HCC.^22^^,^^23^ The anomalous activation of the phosphoinositide 3-kinase (PI3K)/protein kinase B (Akt) signaling pathway and Wnt/β-catenin signaling pathway plays a vital role in the occurrence, progression, and drug resistance of HCC (Fig. 1).^24^, ^25^, ^26^, ^27^ In addition, HBx can interfere with the repair of damaged DNA, driving tumor development.^28^ Importantly, different HBV genotypes and mutations may produce various HBx proteins that participate in the aforementioned processes, resulting in distinct biological outcomes. In addition, the process of HBV evolution into HCC is substantially influenced by the viral load, hepatitis B virus e antigen (HBeAg), and hepatitis B surface antigen (HBsAg). HBV co-infection with hepatitis C virus or hepatitis D virus may also increase the possibility of progression to HCC.^29^

## circRNAs in HBV-induced HCC

**Figure 2:**
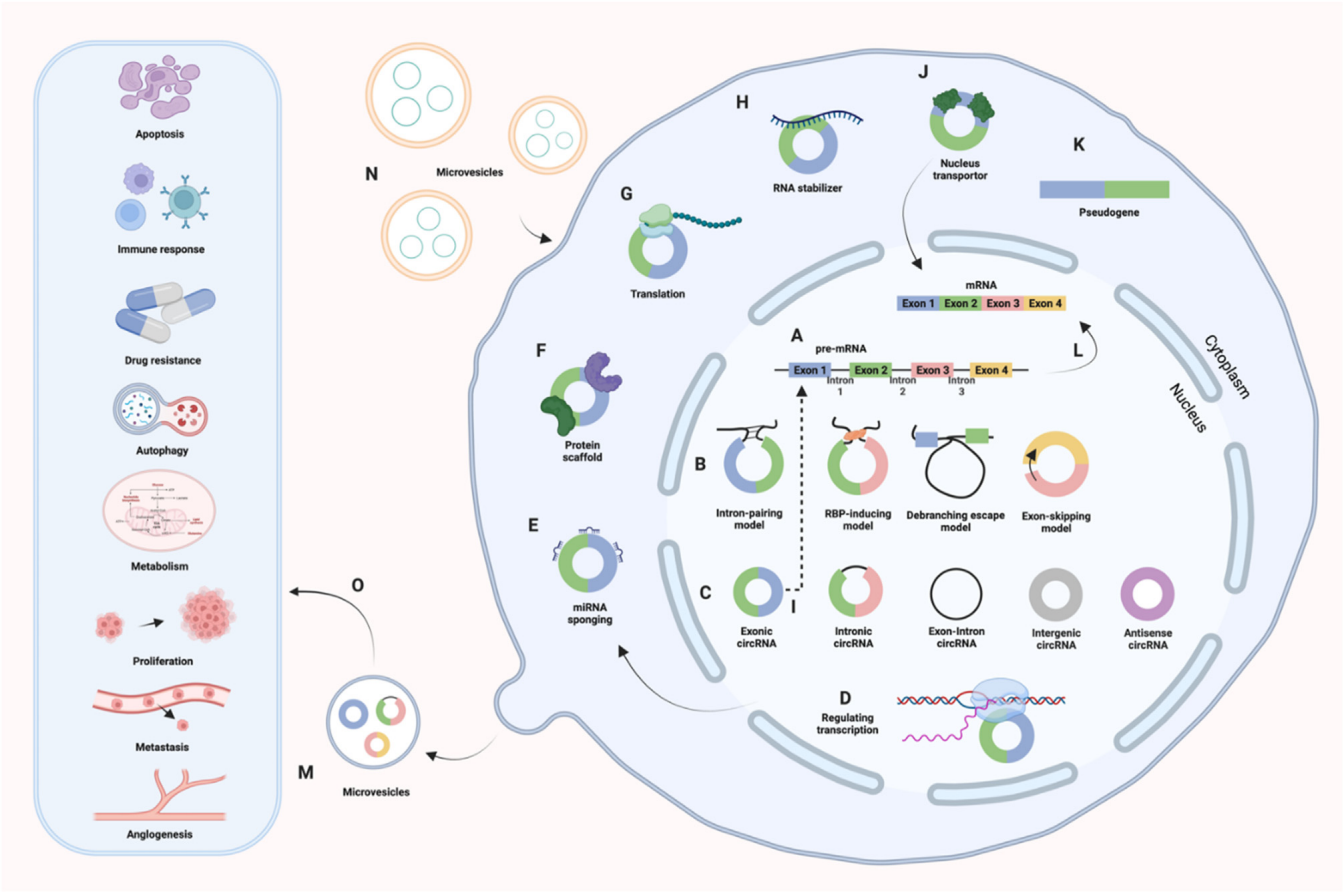
Biogenesis and functions of circRNAs. **(A, B)** Formation mechanisms of circRNAs: intron-pairing model, RNA-binding protein (RBP)-inducing model, debranching escape model, and exon-skipping model. Intron-pairing model: The flanking introns of an exon contain complementary sequences, facilitating back-splicing and circRNA formation by bringing splice sites closer. RBP-inducing model: RBPs bind to flanking intronic sequences and get them in proximity, enabling back-splicing and circularization. Debranching escape model: When a pre-mRNA undergoes splicing to remove introns and join exons, 2′–5′ phosphodiesters at the intron's branch points are conducive to circRNA cyclization. Exon-skipping model: During exon skipping, exons form lariats, producing circRNAs by back-splicing.^11^**(C)** Types of circRNAs: exonic circRNAs, intronic circRNAs, exon-intron circRNAs, intergenic circRNAs, and antisense circRNAs.^6^, ^7^, ^8^**(D**–**K)** The behavior mechanisms of circRNAs: regulating transcription, binding to miRNA, protein scaffolding, translation, producing pseudogenes,^108^ stabilizing RNA molecules, facilitating nucleus transport, and modulating alternative splicing. **(L)** Generation of mRNA from pre-mRNA. **(M, N)** Release and reception of circRNAs via microvesicles. **(O)** Biological functions of circRNAs in cancers.

Advancements in high-throughput sequencing techniques since 2010 have provided researchers with compelling tools to explore circRNAs. Thousands of circRNAs that are differentially expressed in cancer and adjacent normal tissues have been screened using microarrays and next-generation sequencing.^30^^,^^31^ These circRNAs can regulate gene expression by competing with mRNAs for binding sites on miRNAs, interacting with proteins, regulating transcription, influencing alternative splicing, stabilizing RNA molecules, and promoting nuclear transport and translation. These functions performed by circRNAs play significant roles in drug resistance and various pathological changes, including metastasis, apoptosis, invasion, and metabolism (Fig. 2).^32^, ^33^, ^34^, ^35^, ^36^, ^37^, ^38^, ^39^, ^40^, ^41^ Furthermore, circRNAs have emerged as an ideal biomarker for cancer diagnosis and prognosis due to their stability and high levels in body fluids such as plasma, serum, exosomes, saliva, gastric and cerebrospinal fluid, and urine.^42^, ^43^, ^44^ Non-invasive liquid biopsy involving circRNAs is gaining considerable attention. Current research also focuses on circRNAs in exosomes and their interaction with tumor microenvironment components.^45^^,^^46^ However, these areas have been seldom explored in the context of HBV-HCC.

Over the past two decades, the role of circRNAs in liver diseases, including liver cancers, nonalcoholic fatty liver, liver fibrosis, and hepatitis B, has been extensively investigated.^47^, ^48^, ^49^, ^50^ Nevertheless, the primary focus of current research remains on HCC, which is the fifth leading cause of cancer-related deaths worldwide. The studies have demonstrated that circRNAs function as regulators in HCC by regulating drug resistance (anti-PD1, sorafenib, and cisplatin resistance), aerobic glycolysis, and tumorigenesis.^51^, ^52^, ^53^, ^54^ Developing targeted therapies to improve patient survival rates and quality of life necessitates an understanding of the mechanisms of circRNAs in HCC. Meng et al demonstrated that delivering circRNA via nanoparticles showed promise in inhibiting HCC progression without causing apparent toxicity to major organs *in vivo*.^55^ Moreover, early detection of HCC remains a challenge. Increasing research suggests that circRNA may become a desirable biomarker for the diagnosis and prognosis of HCC.^56^ However, further research is needed to identify early HCC biomarkers with high specificity and sensitivity. This could significantly improve the survival rate of HCC patients. Based on the underlying causes leading to HCC, HCC can be classified into the following categories, virus-related, alcohol-related, metabolic-associated fatty liver disease-related, toxin exposure-related, hereditary disease-related, and autoimmune disease-related HCC. It is indeed more beneficial to explore the specific circRNAs in HCC induced by diverse etiological factors for possible early personalization diagnosis and treatment. HBV-induced HCC is considered one of the major types of HCC, particularly in Asia and Africa. The following section discusses some circRNAs involved in the pathogenesis of HBV-induced HCC.

### Hsa-circRNA-104351 and hsa-circRNA-100327

Cui et al used a microarray to analyze circRNA profiles in three pairs of HBV-HCC and matching non-tumor tissues,^57^ identifying 24 up-regulated circRNAs and 23 down-regulated circRNAs. The most up-regulated circRNA was hsa-circRNA-104351, which bound to multiple miRNAs including hsa-miR-490-5p, hsa-miR-876-5p, hsa-miR-619-3p, hsa-miR-311-3p, and hsa-miR-411-3p. Moreover, hsa-circRNA-100327, the most down-regulated circRNA, interacted with hsa-miR-637, hsa-miR-326, hsa-miR-330-5p, hsa-miR-646, and hsa-miR-24-3p. A limitation of this research is that it only focuses on the circRNA expression profiles and circRNA-miRNA networks without exploring their roles in HBV-induced HCC onset and metastasis or their potential as diagnostic markers. Moreover, the differential expression of these circRNAs was not validated in samples or using real-time quantitative PCR.

### circRNA-100338

Huang et al elucidated a ceRNA network functioning in HBV-HCC using circRNA microarray analysis on four pairs of HCC and corresponding non-tumor tissues, identifying 226 differentially expressed circRNAs (189 up-regulated and 37 down-regulated).^58^ They validated circRNA-100338 expression in tumors and adjacent normal tissues from 80 HCC patients with hepatitis B, linking high levels of circRNA-100338 to tumor metastasis. miR-141-3p counteracts the function of circRNA-100338, negatively regulating HBV-HCC metastasis, likely targeting metastasis suppressor 1 (MTSS1). Although acting as a metastatic trigger in this study, MTSS1 commonly inhibits tumor migration through multiple mechanisms, including inhibition of cell migration and invasion, impact on cell adhesion, suppression of cancer angiogenesis, and regulation of signaling pathways. This study identified circRNA-100338 as a novel biomarker for HBV-HCC diagnosis. However, a potential limitation of this study is that the microarray analysis was conducted in HCC rather than in HBV-related HCC, indicating that circRNA-100338 may not be exclusively expressed in HBV-HCC. Therefore, gene ontology (GO) and the Kyoto Encyclopedia of Genes and Genomes (EFGG) pathway analyses are recommended to understand circRNAs' functions better.

### circRNA-101764, 100381, 103489

Using microarrays and quantitative reverse transcription PCR on HCC and matching adjacent normal tissues, Wang et al found 24 up-regulated and 23 down-regulated circRNAs from different sources, with circRNA-101764, 100381, and 103489 being significantly up-regulated.^59^ Subsequently, mRNAs were quantified, followed by GO analysis to illustrate the biological processes, molecular functions, and cellular components associated with the mRNAs. Biological processes were mostly related to the regulation of macromolecular metabolic processes, positive regulation of macromolecular processes, and positive regulation of cellular processes. Cellular components included the intracellular components and nuclear lumen. Protein binding, receptor binding, and kinase regulator activity were assessed in molecular functions. Additionally, KEGG pathway analysis revealed that the PI3K-Akt signaling pathway was highly relevant to the biological processes. Finally, the interaction of the miRNA 181 family with circRNA-101764 was analyzed. These circRNAs could serve as novel biomarkers for the early detection and prognosis of HCC. Increasing the sample size and improving the validation methods are essential for confirming the functions of these circRNA networks in HBV-HCC.

### circRNA-10156

Wang et al employed whole-genome sequencing to screen circRNAs from RNAs extracted from HBV-positive liver tumors and paired pericancerous tissues assessing sequence quality using Q30 indices.^60^ Of the 502 differentially expressed circRNAs, 219 exhibited positive results, and 283 showed unfavorable regulatory functions. Subsequently, GO and KEGG analyses indicated that these circRNAs primarily contributed to cancer progression and metastasis. Subsequently, a complex regulatory network was constructed, and circRNA-10156 was selected to investigate potential mechanisms underlying HBV-HCC onset. circRNA-10156 is generated from the exons of the CGNL1 gene, which encodes an endothelial junction complex protein that can modulate vascular growth. The circRNA-10156/miR-149-3p/Akt1 axis, crucial in HCC cell proliferation regulation, was explored using computational calculations, quantitative reverse transcription PCR, and cell assays. Knockdown of circRNA-10156 up-regulated miR-149-3p levels and decreased Akt1 mRNA level, thereby inhibiting cancer cell proliferation. Akt1 is a protein kinase that phosphorylates various substrate proteins and activates the mammalian target of the rapamycin pathway to stimulate cell growth and proliferation. This study highlights sequence quality and circRNA biogenesis, but a major limitation is the need for validation using large samples.

### circ-ATP5H

Compared with adjacent non-tumor tissues, circ-ATP5H levels were relatively high in HBV-specific cells (*e.g.*, HBV-HCC cells) with miR-138-5p down-regulation. Jiang et al found that miR-138-5p silenced the TNFAIP3 gene by binding to its mRNA, whereas circ-ATP5H promoted TNFAIP3 expression by competitively binding to miR-138-5p.^61^ Furthermore, low levels of TNFAIP3 suppressed HBV replication and HBsAg and HBeAg production, mitigating HCC onset and progression. Thus, interference with circ-ATP5H affects HBV-HCC progression through the circ-ATP5H/miR-138-5p/TNFAIP ceRNA network. TNFAIP3 contributes to antiviral immunity by maintaining immune balance and protecting tissue health by suppressing inflammatory responses, reducing cell apoptosis, and negatively regulating immune signaling pathways like nuclear factor kappa B (NF-κB) signaling pathway. Further studies with larger cohorts are needed to verify the differential expression and function of circRNA-ATP5H.

### circ-ARL3

m6A is the most prevalent chemical modification of RNA, particularly in eukaryotes.^62^ RNA methylation is a reversible process that adds a methyl group to adenosine via a complex of proteins named methyltransferase 3 (METTL3) and methyltransferase 14 (METTL14). This modification influences RNA stability, splicing, transport, and translation. Rao et al indicated that HBx in HBV-HCC increased METTL3 levels to facilitate m6A modification of circ-ARL3.^63^ The m6A reader YTHDC1 recognized and bound to m6A-modified sites on circ-ARL3, enhancing back-splicing and formation, thereby increasing circ-ARL3 levels. Microarray analysis identified circ-ARL3 as a differentially expressed circRNA in HBV-HCC. Functionally, circ-ARL3 can compete with oncogenes, such as WNT2, UBE2T, and MDM2, to bind to miR-1305, strengthening HBV-HCC cell proliferation and metastasis. The novelty of this study lies in the m6A modification of circ-ARL3 induced by HBx, suggesting future research directions on RNA modifications in circRNAs for cancer treatment. In addition, HBV-positive and HBV-negative liver cancer tissues and cells were selected for microarray analysis, better reflecting the specific expression of circARL3 in HBV-HCC.

### circ-RNF13

circ-RNF13 is abundantly expressed in HCC cells, with higher expression levels observed in HBV-HCC using high-throughput sequencing and cell assays. In addition, based on the ratio of expression in the nucleus and cytoplasm, circ-RNF13 was mainly localized in the cytoplasm.^64^ Functionally, circ-RNF13 can bind to miR-424-5p to block TGFB-induced factor homeobox 2 (TGIF2) from binding to miR-424-5p, promoting TGIF2 translation in HBV-induced HCC cells. This accelerates tumor growth, metastasis, and HBV replication, increasing HBsAg and HBeAg expression in HBV-HCC. Chen et al reported that abnormal TGIF1 expression contributed to tumor development and progression but also played a role in the inhibition of cell growth and cancer resistance.^64^ Future studies should explore the association between circRNAs regulating HBV-HCC onset and HBV replication to enable simultaneous treatment of HBV infection and HCC in HBV-HCC patients.

### circRNAs related to HBV-HCC treatment by SB and OD extracts

*Scutellaria barbata* D.Don (SB) and *Oldenlandia diffusa* (Willd.) Roxb (OD) are traditional Chinese herbal medicines with anti-inflammatory, anti-oxidant, anti-cancer, and diuretic properties. Total flavonoids from SB and OD extracts effectively suppress tumorigenesis, progression, metastasis, and HBV activity in HBV-HCC by inducing cell cycle arrest in G0/G1. Yang et al analyzed circRNA expression profiles to identify differentially expressed circRNAs associated with HBV-HCC treatment in groups treated with SB and OD extracts compared with controls.^65^ GO and KEGG pathway analyses were conducted to investigate the functions of circRNAs. The most crucial step was to illustrate the circRNA-miRNA regulatory networks. This novel idea links herbal medicines to the influence of circRNAs on HBV-HCC, providing a promising avenue for circRNA studies. Nevertheless, further investigations are essential to validate and elucidate the molecular interactions and functions implicated in circRNA-miRNA-gene networks.

### circRNA HBV-circ-1

In addition to cell-encoded circRNAs, virus-encoded circRNAs also play a crucial role in virology and oncology. Numerous studies have demonstrated that many viruses could produce circRNAs, which are involved in the tumorigenesis of various cancers. These viruses include herpes simplex virus 1, HBV, Epstein–Barr virus, human papillomavirus, influenza A virus, human immunodeficiency virus, Hantaan virus, Dengue virus, human cytomegalovirus, and Middle East respiratory syndrome coronavirus.^66^^,^^67^ Zhu et al identified HBV-circ-1 by RNA sequencing of hepatocytes expressing HBV and verified its generation from pgRNA and pcRNA of HBV at two junction sites. Microarray results showed that HBV-circ-1 primarily resided in the cytoplasm and was expressed in HBV-HCC tissues at higher levels than in paired normal tissues.^68^ In addition, they reported that HBV-circ-1 could accelerate the HCC cell cycle and inhibit HCC cell apoptosis by detecting Ki67 and cyclin D1, thereby promoting tumor growth, migration, and invasion. This could be attributed to the interaction between HBV-circ-1 and cyclin-dependent kinase 1 (CDK1), a protein related to the cell cycle. Moreover, high levels of HBV circ-1 increased CDK1 expression *in vivo* and *in vitro*. CDK1 is a protein kinase that is crucial for cell cycle regulation, which typically forms an active complex with cyclin B, performing diverse functions at various stages of the cell cycle. These results provide potential insights into the efficacy of therapeutic drugs.

### Hsa-circ-0066966

Microarray analysis revealed that the proportion of differentially expressed circRNAs in HBV-positive HCC cells was higher than in HBV-negative HCC cells, followed by GO and KEGG pathway enrichment analyses to assess the functions of circRNAs. Yuan et al conducted real-time quantitative PCR to screen for hsa-circ-0066966 with the highest absolute value of Log_2_FC.^69^ Log_2_FC represents the ratio of expression between two samples, taking the logarithm of base 2. Generally, an absolute value of log_2_FC >1 is considered the screening criterion for differentially expressed genes. Functionally, hsa_circ_0066966 knockdown suppressed the proliferation and migration of HBV-positive HCC cells, whereas its overexpression in HBV-negative carcinoma cells played a facilitating role. This approach should be improved by considering the effects of the hsa-circ-0066966 regulatory network on HBV-HCC.

### circBACH1

Exons 3 and 4 of the BACH1 gene cyclize to form circBACH1, which is highly expressed in HCC tissues and HBV-related HCC cells. Du et al found that the negative regulation of circBACH1 effectively inhibited HBV replication and HCC metastasis.^70^ During this process, miR-200a-3p acts as a downstream molecule that binds to circBACH1. Computational analysis was then performed to predict the mRNA and construct a complete ceRNA network. Interestingly, mitogen-activated protein kinase kinase kinase 2 (MAP3K2) 3′UTR combines miR-200a-3p competitively with circBACH1. The circBACH1/miR200a-3p/MAP3K2 axis influences the activities of both HBV and HCC, suggesting the potential of circBACH1 as a therapeutic target for simultaneously eliminating HBV cccDNAs and suppressing HBV-HCC development in patients with HBV-HCC.

### circ-0027089

Nucleus accumbens-associated protein 1 (NACC1) has been implicated in various cancers; however, its role in tumorigenesis is complex and varies depending on the cancer type. Microarray analysis revealed that HBV-HCC cells and tissues overexpressing circ-0027089 positively correlated with NACC1 expression. Additionally, He et al found that circ-0027089 could promote tumor growth, inhibit cancer cell apoptosis, and affect cancer metastasis in patients with HBV-HCC. The use of siRNA to interfere with circ-0027089 expression exhibited the opposite effect.^71^ These functions are modulated by the circ-0027089/miR-136-5p/NACC1 mRNA network. circ-0027089 binds to miR-136-5p and inhibits its binding to NACC1 3′UTR, encouraging NACC1 expression.

### circRNA cFAM210A

cFAM210A was the most significantly deregulated circRNA in the HCC cell lines overexpressing the HBx protein and in patients with HBV-induced HCC. Yu et al demonstrated that HBx could increase the expression of the m6A methyltransferase RBM15, which could promote the methylation and subsequent degradation of cFAM210A.^72^ The level of cFAM210A is negatively correlated with the proliferation, stemness, and tumorigenicity of HCC cells. Moreover, the down-regulation of cFAM210A results in less Y-box binding protein 1 (YBX1) binding to cFAM210A. Consequently, more YBX1 was phosphorylated, which could promote tumorigenesis in HCC by enhancing its transactivation function toward mesenchymal–epithelial transition factor (MET). However, Yu et al only focused on the transactivation function of YBX1.^72^ It remains to be explored whether cFAM210A can affect other functions of YBX1 to induce HBV-HCC, such as DNA repair, DNA replication, pre-mRNA splicing, and disassembly of nucleoli.

### circSFMBT2

RNA sequencing was used to find differentially expressed circSFMBT2 between HBx-overexpressing HCC cell lines and non-HBx-overexpressing HCC cell lines. Intriguingly, Liu et al confirmed the component, stability, and intracellular localization of circSFMBT2.^73^ Moreover, low levels of circSFMBT2 in HCC cell lines and tumor tissues are associated with poor prognosis and increased invasion, yet circSFMBT2 was not validated in HBV-HCC cell lines and tumor tissues. circSFMBT2 influences HCC metastasis via the miR-665/TIMP metallopeptidase inhibitor 3 (TIMP3) pathway. HBx down-regulated circSFMBT2 by interacting with DExH-box helicase 9 (DHX9), promoting DHX9 to bind flanking Alu elements, and inhibiting the formation of circSFMBT2.

### circRNA1002

Li et al screened circRNA1002 as a potential biomarker for HBV-HCC.^74^ However, a comprehensive evaluation using receiver operating characteristic curves was not performed to assess the diagnostic performance of circRNA1002. Furthermore, the sensitivity and specificity remain unclear, which undermines its reliability as a potential biomarker. This study primarily focused on the biological functions and pathways identified through GO and KEGG pathway enrichment analyses. RNA sequencing was performed to identify differentially expressed circRNAs between HBV-HCC patients and asymptomatic HBV carriers, and circRNA1002 was confirmed by real-time quantitative PCR. Computational analysis visualized multiple circRNA-miRNA-mRNA regulatory networks, involving nine miRNAs and three mRNAs related to circRNA1002. Future studies should focus on the influence of circRNA1002 networks on the onset and development of HBV-HCC (Table 1).

**Table 1:** Overview of the verified circRNAs in HBV-related hepatocellular carcinoma.

circRNAs	Type (source)	Mechanism			Up/Down	Reference
		Role	Molecular	Potential function		
Hsa-circRNA-104351	Exonic	miRNA sponge	Hsa-miR-490-5p	None	Up	57
			Hsa-miR-876-5p			
			Hsa-miR-619-3p			
			Hsa-miR-311-3p			
			Hsa-miR-411-3p			
Hsa-circRNA-102814	Exonic	miRNA sponge	Hsa-miR-516a-5p	None	Up	
			Hsa-miR-224-5p			
			Hsa-miR-501-5p			
			Hsa-miR-429			
			Hsa-miR-500a-5p			
Hsa-circRNA-103489	Exonic	miRNA sponge	Hsa-miR-654-3p	None	Up	
			Hsa-miR-511-5p			
			Hsa-miR-632			
			Hsa-miR-643			
			Hsa-miR-889-5p			
Hsa-circRNA-102109	Exonic	miRNA sponge	Hsa-miR-1301-3p	None	Up	
			Hsa-miR-20b-3p			
			Hsa-miR-505-5p			
			Hsa-miR-616-3p			
			Hsa-miR-761			
Hsa-circRNA-100381	Exonic	miRNA sponge	Hsa-miR-525-5p	None	Up	
			Hsa-miR-544a			
			Hsa-miR-345-3p			
			Hsa-miR-577			
			Hsa-miR-587			
Hsa-circRNA-100327	Exonic	miRNA sponge	Hsa-miR-637	None	Down	
			Hsa-miR-326			
			Hsa-miR-330-5p			
			Hsa-miR-646			
			Hsa-miR-24-3p			
Hsa-circRNA-101764	Exonic	miRNA sponge	Hsa-miR-181b-5p	None	Down	
			Hsa-miR-181c-5p			
			Hsa-miR-181d-5p			
			Hsa-miR-181a-5p			
			Hsa-miR-329-5p			
Hsa-circRNA-101092	Exonic	miRNA sponge	Hsa-miR-631	None	Down	
			Hsa-miR-612			
			Hsa-miR-221-5p			
			Hsa-miR-889-3p			
			Hsa-miR-1298-3p			
Hsa-circRNA-001225	Intronic	miRNA sponge	Hsa-miR-30b-3p	None	Down	
			Hsa-miR-887-5p			
			Hsa-miR-26b-3p			
			Hsa-miR-485-5p			
			Hsa-miR-363-5p			
Hsa-circRNA-102904	Exonic	miRNA sponge	Hsa-miR-519c-3p	None	Down	
			Hsa-miR-148a-3p			
			Hsa-miR-519a-3p			
			Hsa-miR-567			
			Hsa-miR-205-3p			
Hsa-circRNA-104075	Unknown	Unknown	Unknown	Promoting cell proliferation while inhibiting apoptosis and autophagy^103^	Up	58
Hsa-circRNA-100338	Unknown	miRNA sponge	miR-141-3p	Enhancing the migratory and invasive capacities of tumor cells	Up	
Hsa-circRNA-101764	Unknown	miRNA sponge	miR-181b-5p miR-181c-5p miR-181d-5p miR-181a-5p miR-329-5p	None	Up	59
Hsa-circRNA-100381	Unknown	miRNA sponge	miR-525-5p miR-544a miR-345-3p miR-577 miR-587	None	Up	
Hsa-circRNA-103489	Unknown	miRNA sponge	miR-654-3p miR-511-5p miR-632 miR-643 miR-889-5p	None	Up	
circRNA-10156	Exonic (CGNL1 gene exons)	miRNA sponge	miR-149-3p	Facilitating tumor cell proliferation	Up	60
circ-ATP5H	Unknown	miRNA sponge	miR-138-5p	Inducing HBV replication and expression	Up	61
circ-ARL3	Unknown	miRNA sponge	miR-1035	Promoting the proliferation and invasion of HBV^+^ HCC cells	Up	63
circ-RNF13	Unknown	miRNA sponge	miR-424-5p	Promoting tumor cell proliferation, colony formation, migration, invasion, and HBV replication; increasing the levels of HBsAg and HBeAg	Up	64
Hsa-circ-0006508	Unknown	miRNA sponge	Unknown	Regulating cell proliferation, migration, metastasis, and glycolysis^104^	Unknown	65
Hsa-circ-0100212	Unknown	miRNA sponge	Unknown	None	Unknown	
Hsa-circ-0001725	Unknown	miRNA sponge	Unknown	Influencing cell proliferation and tumorigenicity^105^	Unknown	
Hsa-circ-0020028	Unknown	miRNA sponge	Unknown	None	Unknown	
Hsa-circ-0000643	Unknown	miRNA sponge	Unknown	Regulating cancer ferroptosis and progression^106^	Unknown	
Hsa-circ-044177	Unknown	miRNA sponge	Unknown	None	Unknown	
circRNA HBV-circ-1	Unknown (HBV pgRNA/pcRNA)	Protein scaffold	CDK1	Enhancing tumor cell proliferation, migration, and invasion while inhibiting apoptosis	Up	68
Hsa-circ-0066966	Unknown (GOLGB1)	miRNA sponge	Hsa-miR-214-3p	Enhancing the proliferation and migration of HBV-positive liver cancer cells	Up	69
			Hsa-miR-922			
			Hsa-miR-646			
			Hsa-miR-3619-5p			
			Hsa-miR-374a-3p			
Hsa-circ-0079954	Unknown (HECW1)	miRNA sponge	Hsa-miR-383-3p	None	Up	
			Hsa-miR-7161-3p			
			Hsa-miR-3065-3p			
			Hsa-miR-127-5p			
			Hsa-miR-4291			
Hsa-circ-0060534	Unknown (SLPI)	miRNA sponge	Hsa-miR-4480	None	Up	
			Hsa-miR-6791-3p			
			Hsa-miR-4776-5p			
			Hsa-miR-6165			
			Hsa-miR-664a-3p			
Hsa-circ-0088524	Unknown (GOLGA1)	miRNA sponge	Hsa-miR-3689e	None	Up	
			Hsa-miR-6740-3p			
			Hsa-miR-6809-3p			
			Hsa-miR-3619-5p			
			Hsa-miR-3689b-5p			
Hsa-circ-0062852	Unknown (MORC2)	miRNA sponge	Hsa-miR-4277	None	Down	
			Hsa-miR-514b-3p			
			Hsa-miR-584-3p			
			Hsa-miR-514a-3p			
			Hsa-miR-409-3p			
Hsa-circ-0030525	Unknown (MYCBP2)	miRNA sponge	Hsa-miR-3918	None	Down	
			Hsa-miR-487a-5p			
			Hsa-miR-27b-3p			
			Hsa-miR-1273g-5p			
			Hsa-miR-199a-5p			
Hsa-circ-0032138	Unknown (HIF1A)	miRNA sponge	Hsa-miR-5708	Influencing cell proliferation and stem cell features^107^	Down	
			Hsa-miR-22-5p			
			Hsa-miR-6504-5p			
			Hsa-miR-664a-5p			
			Hsa-miR-149-5p			
Hsa-circ-0085289	Unknown (CTHRC1)	miRNA sponge	Hsa-miR-338-3p	None	Down	
			Hsa-miR-29a-3p			
			Hsa-miR-5586-5p			
			Hsa-miR-6507-3p			
			Hsa-miR-29c-3p			
Hsa-circ-0091095	Unknown (ATRX)	miRNA sponge	Hsa-miR-377-3p	None	Down	
			Hsa-miR-3133			
			Hsa-miR-4511			
			Hsa-miR-5195-3p			
			Hsa-miR-145-5p			
circBACH1	Exonic (BACH1)	miRNA sponge	miR-200a-3p	Promoting HBV replication, proliferation, and metastasis in HBV-transfected hepatoma cells, thereby contributing to hepatoma progression	Up	70
circ-0027089	Unknown	miRNA sponge	miR-136-5p	Promoting tumor cell proliferation, migration, and invasion while inhibiting cell cycle arrest and apoptosis	Up	71
Hsa_circ_0003979	Exonic (FAM210A)	Protein sponge	YBX1	Reducing the proliferation, stemness, and tumorigenicity of HBx-induced HCC cells	Down	72
Hsa_circ-0000211	Exonic (SFMBT2)	miRNA sponge	miR-665	Inhibiting tumor metastasis, vascular invasion, and capsule invasion	Down	73
circRNA1002	Unknown (MED13L)	miRNA sponge	Hsa-miR-181b-5p Hsa-miR-181d-5p Hsa-miR-2116-5p Hsa-miR-3119 Hsa-miR-4423-3p Hsa-miR-4709-3p Hsa-miR-4752 Hsa-miR-503-5p Hsa-miR-892b	None	Down	74
circRNA7941	Unknown (MBOAT2)	Unknown	Unknown	None	Down	
circRNA44142	Unknown (PPP2R5C)	Unknown	Unknown	None	Down	
circRNA26265	Unknown (CDC73)	Unknown	Unknown	None	Down	
circRNA26796	Unknown (KDM6A)	Unknown	Unknown	None	Down	
circRNA4910	Unknown (NRIP1)	Unknown	Unknown	None	Down	
circRNA39338	Unknown (AKT3)	Unknown	Unknown	None	Down	
circRNA364	Unknown (gTF2F2)	Unknown	Unknown	None	Down	
circRNA36484	Unknown (BRE)	Unknown	Unknown	None	Down	
circRNA7935	Unknown (LINC00299)	Unknown	Unknown	None	Down	
circRNA35761	Unknown (PARP8)	Unknown	Unknown	None	Down	

### circRNAs as biomarkers for HBV-related HCC

HBV-HCC is the most prevalent type of HCC, is primarily asymptomatic in the early stages, and lacks effective diagnostic methods. Treatment outcomes for advanced HBV-HCC are usually poor. Several blood-based biomarkers, such as alpha-fetoprotein (AFP), alpha-l-fucosidase (AFU), and des-gamma-carboxy prothrombin (DCP), have been used for the early detection of HCC over the past decades; however, these biomarkers are neither sensitive nor specific.^75^ Therefore, identifying biomarkers with high specificity and sensitivity is urgently needed. A liquid biopsy is a non-invasive procedure for determining disease status using body fluids such as blood, urine, and saliva. circRNAs have recently gained attention in the context of liquid tumor biopsies, with the potential to be useful diagnostic and prognostic indicators for HBV-HCC.

Yu et al identified hsa-circ-0000976, hsa-circ-0007750, and hsa-circ-0139897, which were assembled into circPanel using microarray analysis.^76^ This study utilized three sets of biomarkers, circPanel, AFP, and circPanel plus AFP, to diagnose HBV-HCC, chronic hepatitis B, and HBV-cirrhosis, with a set of healthy controls. The results showed that the area under the curves (AUCs) of the circPanel group were similar to those in the circPanel plus AFP group and higher than the AUCs of the AFP group. All AUCs in the circPanel group were over 0.80. The performance of circPanel was excellent in discriminating HBV-HCC, including small HCC, AFP-negative HCC, and AFP-negative small HCC, from other diseases and healthy controls. However, the need for a group that focuses on individuals with early HBV-HCC is a major limitation of this study.

Zhu et al performed microarray analysis and quantitative reverse transcription PCR to detect circ-0027089, which exhibited remarkable differential expression in plasma samples obtained from patients with HBV-HCC and HBV-cirrhosis.^77^ However, the AUCs of circ-0027089 were consistently lower than those of AFP, and the sensitivity and specificity of circ-0027089 plus AFP were poor for diagnosing HBV-HCC. Additionally, it was postulated that circ-0027089 could regulate the development and metastasis of HBV-HCC by predicting the target miRNAs.

Tumors and non-tumor tissues from 86 HBV-HCC patients were collected to identify hsa_circ_0004018 and hsa_circ_0003570 by quantitative reverse transcription PCR. Kang et al divided all patients into the high-expression group and low-expression group according to the median values.^78^ Based on the Kaplan–Meier curve, overall survival and progression-free survival were significantly different in the hsa_circ_0003570 expression group, but not in the hsa_circ_0004018 expression group. Higher hsa_circ_0003570 expression was linked to worse overall survival in the high expression group. Moreover, Kang et al found that the combination of hsa_circ_0004018 and hsa_circ_0003570 showed better overall survival and progression-free survival than the non-combination group, but the difference was not significant. Kang et al correlated the expression levels of circRNAs with clinical information such as age, liver function profiles, TNM stages, and body composition profiles. The AUC of the receiver operating characteristic curve for both circRNAs in predicting survival outcome was not significant (0.586 in hsa_circ_004018 and 0.619 in hsa_circ_0003570).^78^

Three circRNAs in the HCC group were significantly up-regulated compared with the CH group, healthy group, and HCC post-operation group. Wu et al validated these circRNAs in the training and validation cohorts, using receiver operating characteristic curves to assess the sensitivity and specificity for HCC diagnosis and prognosis.^79^ The AUC of circ_0009582, circ_0037120, and circ_0140117, a combination of circRNAs (factors), AFP, and a combination of circRNAs and AFP (merged) were 0.688, 0.742. 0.762, 0.800, 0.740, and 0.988, respectively, in the training set. Similar results were obtained in the validation set. Moreover, the expression levels of these circRNAs in plasma samples positively correlated with the expression levels in tumor samples. Wu et al incubated circRNAs at room temperature for a few hours and conducted several freeze–thaw cycles to validate their stability in human body fluid.^79^

Finally, microarray analysis and quantitative reverse transcription PCR were used to identify the serum exosomal circRNA called hsa-circ-0028861. All AUCs of circ-0028861 were approximately 0.80 in differentiating between HBV-HCC, small HBV-HCC, early HBV-HCC, and AFP-negative HBV-HCC from hepatitis B infection and HBV-cirrhosis. Sensitivity and specificity were approximately 70 % and 80 %, respectively. Furthermore, the AUCs of circ-0028861 plus AFP (0.91, 0.82, and 0.86, respectively) were higher than those of AFP (0.82, 0.69, and 0.76, respectively) for distinguishing HBV-HCC from chronic HBV infection, HBV without cirrhosis, and hepatitis B with cirrhosis. Hsa-circ-0028861 exhibited good diagnostic performance, particularly when combined with AFP. Additionally, Wang et al predicted the target miRNAs and performed pathway enrichment analysis to demonstrate that hsa-circ-0028861 may influence HBV-HCC progression (Table 2).^80^

**Table 2:** Performance of several circRNAs and AFP based on existing literature on HBV-HCC diagnosis.

Groups	Biomarkers	AUC (95 % confidence interval)	Sensitivity (%)	Specificity (%)	Reference
HCC *vs*. non-HCC	circPanel	0.864 (0.830–0.898)	85.5	87.3	76
	AFP	0.769 (0.728–0.810)	65.2	88.6	
	circPanel plus AFP	0.874 (0.840–0.907)	91.7	83.1	
HCC *vs*. healthy control	circPanel	0.875 (0.829–0.921)	85.5	89.5	
	AFP	0.819 (0.777–0.862)	65.2	98.7	
	circPanel plus AFP	0.906 (0.862–0.950)	91.7	89.5	
HCC *vs*. chronic hepatitis B	circPanel	0.859 (0.809–0.908)	85.5	86.3	
	AFP	0.732 (0.672–0.792)	65.2	81.3	
	circPanel plus AFP	0.846 (0.789–0.903)	91.7	77.5	
HCC *vs*. cirrhosis	circPanel	0.859 (0.809–0.908)	85.5	86.3	
	AFP	0.757 (0.701–0.813)	65.2	86.3	
	circPanel plus AFP	0.871 (0.819–0.923)	91.7	82.5	
Small HCC *vs*. non-HCC	circPanel	0.851 (0.799–0.903)	83.0	87.3	
	AFP	0.738 (0.671–0.805)	59.1	88.6	
	circPanel plus AFP	0.864 (0.818–0.910)	89.8	83.1	
Small HCC *vs*. healthy control	circPanel	0.862 (0.801–0.923)	83.0	89.5	
	AFP	0.789 (0.718–0.860)	59.1	98.7	
	circPanel plus AFP	0.896 (0.842–0.950)	89.8	89.5	
Small HCC *vs*. chronic hepatitis B	circPanel	0.846 (0.783–0.909)	83.0	86.3	
	AFP	0.702 (0.622–0.782)	59.1	81.3	
	circPanel plus AFP	0.836 (0.771–0.902)	89.8	77.5	
Small HCC *vs*. cirrhosis	circPanel	0.846 (0.783–0.909)	83.0	86.3	
	AFP	0.727 (0.649–0.804)	59.1	86.3	
	circPanel plus AFP	0.861 (0.800–0.922)	89.8	82.5	
HCC *vs*. non-HCC	Hsa-circ-0027089	0.784	57.81	84.80	77
	AFP	0.857	68.75	95.50	
HCC *vs*. healthy control	Hsa-circ-0027089	0.794	57.81	85.00	
	AFP	0.873	68.75	100.00	
HCC *vs*. cirrhosis	Hsa-circ-0027089	0.765	57.81	85.00	
	AFP	0.829	68.75	87.50	
HCC *vs*. healthy control or chronic hepatitis	circ-0009582	0.805 (0.718–0.891)	Unknown	Unknown	79
	circ-0037120	0.835 (0.748–0.922)	Unknown	Unknown	
	circ-0140117	0.845 (0.766–0.924)	Unknown	Unknown	
	Factors	0.857 (0.713–0.940)	Unknown	Unknown	
	AFP	0.803 (0.713–0.892)	Unknown	Unknown	
	Merged	0.955 (0.915–0.994)	Unknown	Unknown	
HCC *vs*. HBV plus cirrhosis	Hsa-circ-0028861	0.79 (0.72–0.87)	67.86	82.69	80
	Hsa-circ-0028861 plus AFP	0.86 (0.80–0.93)	76.36	86.27	
	AFP	0.76 (0.68–0.84)	43.64	96.08	
HCC *vs*. HBV	Hsa-circ-0028861	0.83 (0.75–0.91)	76.79	78.95	
	Hsa-circ-0028861 plus AFP	0.91 (0.85–0.96)	81.82	89.09	
	AFP	0.82 (0.74–0.90)	78.18	72.73	
HCC *vs*. cirrhosis	Hsa-circ-0028861	0.75 (0.66–0.85)	67.86	76.60	
	Hsa-circ-0028861 plus AFP	0.82 (0.74–0.91)	76.36	80.85	
	AFP	0.69 (0.59–0.79)	38.18	97.87	
Small HCC *vs*. HBV plus cirrhosis	Hsa-circ-0028861	0.81 (0.73–0.89)	70.00	80.77	
Stage 1 or 2 HCC *vs*. HBV plus cirrhosis	Hsa-circ-0028861	0.82 (0.74–0.91)	71.43	82.69	

Note: HCC, hepatocellular carcinoma; AFP, alpha-fetoprotein; HBV, hepatitis B virus; AUC, area under the curve.

## Methodology of circRNA research in HBV-HCC

**Figure 3:**
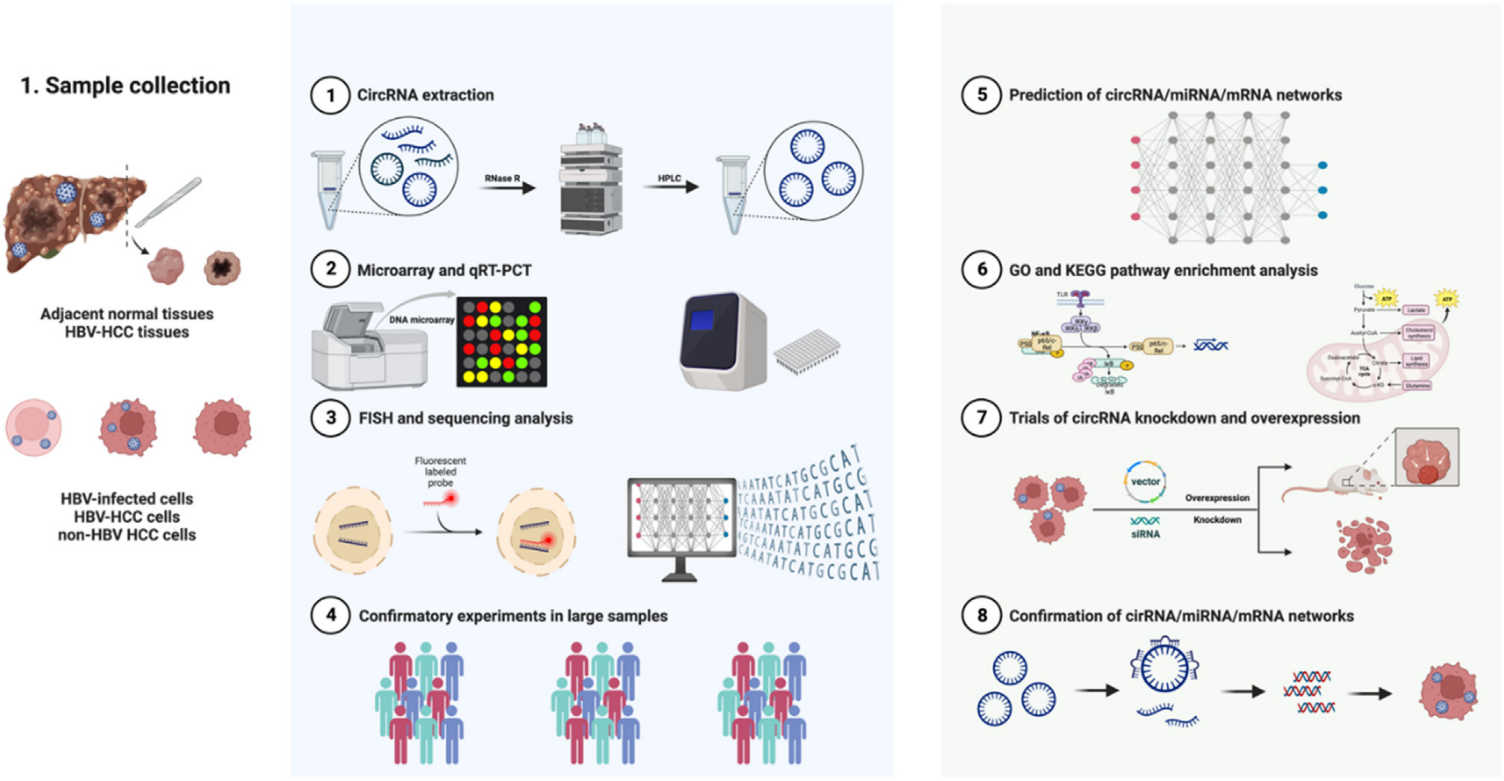
Flow chart of the study exploring the functions of circRNAs in HBV-HCC: Sample collection from HBV-HCC tissues and adjacent normal tissues with HBV or from HBV-infected cells, HBV-HCC cells, and non-HBV HCC cells. **(1)** Extraction of total RNAs from samples and purification of circRNAs by RNase R digestion and high-performance liquid chromatography (HPLC). **(2)** Analysis and verification of differentially expressed circRNAs by microarray and quantitative reverse transcription PCR. **(3)** circRNA localization by fluorescence *in situ* hybridization (FISH) and sequencing analysis. **(4)** Verification of circRNAs in large samples. **(5, 6)** Prediction of ceRNA networks by computers and functions of circRNAs with GO and KEGG pathway enrichment analysis. **(7, 8)** Cell and animal assays were performed to confirm the ceRNA network and function of circRNAs. HCC, hepatocellular carcinoma; HBV, hepatitis B virus.

Research methodologies require further improvement to advance circRNA research in HBV-HCC and identify effective therapeutic targets. Here, we have outlined and analyzed existing methodologies from multiple perspectives, aiming to provide preliminary insights and potential directions for future studies (Fig. 3). Participant selection and sample collection are crucial for successfully identifying HBV-HCC-specific circRNAs. To ensure more convincing results, samples should include HBV-HCC tissues, paired non-tumorous tissues of HBV-HCC patients, and non-HBV-HCC tissues. Before identifying circRNAs, performing RNase R digestion and high-performance liquid chromatography purification of the extracted total RNAs are essential to remove ribosomal and linear RNAs, enriching circRNAs and ensuring the analysis accuracy.

The primary methodologies for identifying circRNAs are microarrays, quantitative reverse transcription PCR, and RNA sequencing, each with its unique advantages and limitations. Microarrays are ideal for the high-throughput detection of known circRNAs at a moderate cost. They are superior in detecting low-expression circRNAs as well as providing comprehensive functional annotation of circRNAs, yet they may miss novel circRNAs, while quantitative reverse transcription RCR is commonly used for validation and quantifying circRNAs in small sample sizes. Additionally, quantitative reverse transcription PCR can be used to discover whether a circRNA that is differentially expressed between lung cancer and matched non-tumorous tissues has similar functions in liver cancer. However, quantitative reverse transcription PCR is not suitable for whole-genome exploration, as only a small number of target circRNAs can be analyzed at a time. RNA sequencing, the most sensitive but also the most expensive method, enables the high-throughput detection of both known and novel circRNAs. RNA sequencing can provide detailed sequence, structure, and potential functional information after complex data analysis. This provides a way for a thorough exploration of circRNAs.

Identified circRNAs will be validated in HBV-HCC, HBV-infected, and non-HBV-HCC liver cell lines. RNA analysis on HBV-infected or HBx-expressing liver cancer cell lines was performed to confirm the circRNAs associated with HBV.^68^ Following sequencing, circRNAs can be analyzed to determine their sources, lengths, types, and circular structures, with localization visualized using fluorescence *in situ* hybridization. Intriguingly, these differentially expressed circRNAs may be derived from HBV rather than the cells.^67^ Moreover, circRNA stability will be assessed by several freeze–thaw cycles. Performing confirmatory trials on large samples can enhance the reliability of our investigations. The prediction of the circRNA/miRNA/mRNA networks is followed by GO and KEGG pathway enrichment analyses that are used to identify the potential functions of circRNAs. An appropriate circRNA-to-miRNA and circRNA-to-protein ratio is a prerequisite for circRNAs to function. Subsequently, knockdown and overexpression experiments were performed on cell lines and mouse models to verify the ceRNA networks and their functions.

Several issues need to be considered in the experimental approaches to exploring HBV-HCC biomarkers. Ideally, the patient cohort should include three groups comprising patients with HBV infection without HCC, non-HBV-HCC, and HBV-HCC. Patients in the HBV-HCC group must be strictly divided into an early HBV-HCC group and an advanced HBV-HCC group based on the patient's clinical features and a uniform scoring system, such as the World Health Organization (WHO) or Edmondson-Steiner grading system. AFP can be used for comparison with circRNAs, as AFP remains a highly reliable index for liver cancer identification. Data for evaluating biomarkers should include calculations of the positive predictive value, negative predictive value, and AUC.

## circRNAs as potential therapeutic targets and therapeutic molecules

circRNAs serve as important molecular targets in cancer treatment. Although these studies are currently at a preliminary stage and have not been effectively implemented in clinical practice, the continuous emergence of new studies indicates increased attention to circRNAs in cancer treatment. The three primary approaches for managing circRNAs associated with tumor therapy are as follows: First, RNA interference (RNAi), which involves three RNA molecules (siRNA, shRNA, and antisense oligonucleotides), is used to silence endogenous circRNAs.^81^^,^^82^ However, RNAi molecules have several disadvantages, including instability, low targeting efficiency, difficult transfection, and immunogenicity. Second, circRNA expression vectors are standard ways of overexpressing circRNAs. The construction of circRNA expression vectors typically involves three cyclization mechanisms, namely, complementary sequence-mediated RNA cyclization, RBP Quaking RNA-binding motifs, and tRNA-based splicing mechanisms. Third, cancer care can be achieved by delivering circRNAs that are synthesized and purified *in vitro*. Currently, chemistry-based, enzyme-based, and ribozyme-based approaches have been used to synthesize circRNAs *in vitro*, and high-performance liquid chromatography is an excellent method for purifying circRNAs after RNase R digestion.^83^, ^84^, ^85^ However, mass production of synthetic circRNAs with immunogenicity *in vivo* is challenging.^86^ Furthermore, the use of ligases in circRNA synthesis unavoidably results in the formation of unwanted byproducts that can induce unpredictable adverse reactions *in vivo*.

Intriguingly, circRNAs have attracted considerable attention in RNA vaccine development because of their excellent stability and ability to encode proteins. Engineered circRNAs are effective templates for protein translation. This process relies on internal ribosome entry sites or m6A modifications.^87^ Moreover, optimizing the constituent elements of circRNA vaccines and RNA modifications can enhance cyclization efficiency, maximize circRNA translation, and reduce circRNA immunogenicity. For example, the m6A modification can eliminate circRNA immunogenicity and enable the production of higher and longer-lasting antigens, thereby neutralizing a higher proportion of antibodies. In addition, the translation efficiency and durability of synthetic circRNAs may be enhanced by optimizing vector topology, 5′ and 3′ untranslated regions, internal ribosome entry sites, and aptamers for translation initiation *in vitro*.^88^ Another advantage of circRNAs in the vaccine field is that they can activate various immune cells and influence different immune pathways.^89^, ^90^, ^91^ Therefore, circRNAs can be used as autoimmune regulatory self-adjuvants to boost antitumor immunity, interfere with the tumor microenvironment, increase vaccination efficacy, and reduce the adverse effects of other immune adjuvants.^92^

These treatment schemes are based on precise delivery systems. Nanoparticles are widely used for circRNA delivery, predominantly lipid nanoparticles, owing to their relatively simple production and stable nanostructures. However, they have limited efficacy in releasing circRNAs. In addition, polymer nanoparticles (*e.g.*, nanocapsules) and inorganic nanoparticles (*e.g.*, gold nanoparticles) are available.^93^^,^^94^ Second, exosomes have emerged as promising endogenous circRNA carriers *in vivo*. They have favorable attributes, such as excellent biocompatibility, low toxicity, and strong designability. Numerous cells in the body generate exosomes, among which cancer-derived exosomes have better tumor-targeting effects. However, mass production and accurate targeting of cancer cells remain challenging. Intriguingly, the investigation of plant-derived exosomes is underway.^95^^,^^96^ Third, virus-like particles and viral vector-mediated circRNA delivery are the standard measures. Virus-like particles are composed of viral structural proteins, such as infectious viruses, with no viral nucleic acids, which are safe and biocompatible. However, this process involves a series of laborious purification procedures. Alternatively, lentiviruses, adenoviruses, and adeno-associated viruses can act as viral expression vectors to indirectly deliver circRNAs.^97^^,^^98^

## Discussion and conclusion

Technological advancements, coupled with the need to develop novel therapeutic approaches for tumors, have prompted more researchers to focus on the role of circRNAs in HBV-HCC. Nevertheless, the present investigation of circRNAs in HBV-HCC is at a rudimentary stage and has many shortcomings. Therefore, this review aims to discuss and analyze existing research on circRNAs in HBV-HCC from multiple perspectives and provide a comprehensive analysis of the procedures and directions for future circRNA research in HBV-HCC. This will be conducive to an in-depth study of circRNAs in HBV-HCC, advancing the diagnosis and treatment of HBV-HCC patients.

First, existing research on circRNAs in HBV-HCC has several limitations, including the need for further investigation of the signaling pathways and circRNAs that regulate translation and transcription functions. Moreover, the role of exosomal circRNAs in HBV-HCC needs to be further explored. In the future, additional exploration of circRNA/miRNA/mRNA networks and circRNAs with the capability to translate polypeptides and scaffold proteins is needed. Further research is warranted to elucidate the downstream signaling pathways associated with the evolution of HBV infection into HCC. Likewise, the role of circRNAs in angiogenesis, immune microenvironment, drug resistance, and immune evasion in HBV-HCC needs further exploration. Programmed cell death ligand 1 (PD-L1) on the surface of tumor cells binds to programmed death-1 (PD-1) on the surface of T cells to inhibit anti-tumor immunity, thereby promoting tumor immune escape. Multiple immune checkpoint inhibitors have been used to treat tumor immune escape, and circRNAs can affect this phenomenon.^99^ In addition, chemical modifications of RNA and HBV products such as HBx, HBeAg, and HBsAg may affect circRNAs by accelerating the onset and progression of HBV-HCC, which presents an area for further research.^63^^,^^73^ Current mainstream research on circRNAs has focused on up-regulated circRNAs; however, down-regulated circRNAs are also critical for disease development and treatment.^100^

To date, diagnostic and prognostic circRNA biomarkers that are sensitive and specific for HBV-HCC remain unknown. An effective circRNA biomarker should be able to distinguish between patients with advanced HBV-HCC, early HBV-HCC, HCC-negative HBV-cirrhosis, and non-cirrhotic, HCC-negative hepatitis B. In addition, the combination of circRNAs and AFP as biomarkers has a higher AUC than that of circRNAs alone.^79^ This provides additional benefits by combining circRNAs with other indicators to improve the sensitivity and specificity of diagnostic biomarkers. Identifying highly sensitive and specific circRNAs as diagnostic biomarkers for early HBV-HCC or as indicators of HBV progression into HCC is crucial to improving patient survival rates and the overall prognosis by identifying those who are at higher risk, thereby initiating more aggressive therapies at an earlier stage. As circRNAs are stable in various body fluids, it is also feasible to investigate the potential of circRNA biomarkers associated with HBV-HCC in urine and saliva.

Moreover, circRNA-related therapies for HBV-HCC, particularly circRNA vaccines, are urgently needed because of the high mortality rate. However, the key obstacles include the inability to precisely target the lesions, enhancing the translational ability of circRNAs, reducing circRNA immunogenicity, and constructing effective circRNA delivery pathways. Among the three previously mentioned delivery pathways, lipid nanoparticles have a unique propensity for the liver tissue and are promising delivery systems for circRNAs to treat HBV-HCC. Interestingly, novel lipid nanoparticles have been developed to improve targeting efficiency and epigenetic modifications of circRNAs have been conducted to improve translational capacity and diminish immunogenicity.^101^^,^^102^ In addition, topical injection may offer a simple and cost-effective delivery strategy for circRNA vaccines owing to the high stability of circRNAs. Nevertheless, relevant research has yet to be conducted. Furthermore, it is worth considering whether selected herbs exhibit anti-tumor properties through the regulation of endogenous circRNAs.^65^

The role of circRNAs in HBV-HCC has been recently documented.^102^ However, in contrast to other previous studies, our review provides a more comprehensive overview of circRNAs that could guide future research on circRNAs in HBV-HCC. Furthermore, the applications of circRNAs as therapeutic targets, therapeutic molecules, and delivery measures are summarized, which will considerably assist in the research of circRNAs within the field of HBV-HCC therapeutics. Finally, suggestions for improving the methodologies of circRNA research have been discussed. The construction of the visual flowchart provides a helpful reference for future research. In general, the functions of circRNAs in HBV-HCC remain poorly understood and less applied in clinical practice, presenting ample opportunities for further exploration. Furthermore, the mechanisms of onset and development may help distinguish between different types of HCC, and the study of circRNAs in HBV-HCC may help facilitate strategizing personalized treatment approaches and precise diagnosis of early HBV-HCC.

## Funding

N.M. sourced for the funding of the manuscript (Universiti Sains Malaysia 1001.PPSP.8012343).

## CRediT authorship contribution statement

**Wenjun Quan:** Writing – review & editing, Writing – original draft, Software, Resources, Project administration, Methodology, Formal analysis, Data curation, Conceptualization. **Kizito Eneye Bello:** Writing – original draft, Conceptualization. **Rafidah Hanim Shueb:** Writing – review & editing, Supervision. **Nazri Mustaffa:** Writing – review & editing, Supervision, Investigation, Funding acquisition.

## Conflict of interests

The authors declared no competing interests.
